# A New Reduction Mastopexy Design for Young Women: Snowman Pattern

**Published:** 2015-07

**Authors:** Yakup Cil, Atacan Emre Kocman

**Affiliations:** 1 Diyarbakır Military Hospital, Department of Plastic Surgery 21000 Diyarbakır, Turkey;; 2 Assistant Professor, Department of Plastic, Reconstructive and Aesthetic Surgery, Eskisehir Osmangazi University Medical School, Eskisehir, Turkey

**Keywords:** Breast reduction, Mastopexy, Snowman design

## Abstract

**BACKGROUND:**

Many young women are satisfied with their large breasts but suffer from sagging due to heaviness. In this article; we present a novel modification of vertical scar breast reduction based on a special indication.

**METHODS:**

From January 2006 to May 2012, twenty five individual patients underwent operation using modified technique with superior pedicle and vertical scar. Young women between ages 25-35 years with voluminous breasts who requested mastopexy rather than reduction were selected for the surgery.

**RESULTS:**

The mean patient age was 30 years and body mass index (BMI) was 27.8±1.07 kg/m^2^. Mean nipple transposition was 6.5 cm. Mean weight for resected tissue was 415 g for left and 419 g for right breast. Mean operative time was 125 minutes. Patients were followed up for 9-22 months. No serious complications encountered in consecutive patient series. The only complication was permanent wrinkling probably due to vertical closure in 5 of 25 patients which did not resolve during the follow-up period.

**CONCLUSION:**

We recommend that the Snowman design is a useful tool for superior pedicle breast reduction technique providing good projection and a short scar in selected patients.

## INTRODUCTION

Breasts are regarded as the expression of feminity and sexual appeal. Thus having large breasts are very important especially for young women. Many young women are usually satisfied with their large breasts, however; some complains of slight ptosis, which is an aesthetic concern rather than physical. Most of them use uplift brassieres or corsettes, to camouflage ptosis. But these conservative methods do not help permanent shaping. So they are motivated for mastopexy and breast reduction surgery.^1^

Breast reduction is one of the most performed operations in plastic surgery practice. Although breast reduction with inverted T scar is the technique of choice because of reliability among the plastic surgeons,^1^ vertical scar breast reduction which enables less scar and longer lasting results, is also welcomed.^2^^,^^3^ The evolution and history of the vertical scar breast reduction and mastopexy is well described in the literature.^4^ The technique was first introduced by Dartique and further developed and popularised by Lassus and Lejour. The learning curve was simplified by Hall-Findlay. Since then, many contributions to the vertical scar breast reduction were reported by various authors.^5^ The dynamics and the principles of the technique were also well established at the same time.^6^ While fullfilling the need of more patients, it also gained increasing popularity among surgeons, recently.^1^^-^^3^

In the present article a novel modification of vertical scar breast reduction based on mastopexy with limited inferior pole resection was described. Young patients with large but mild ptotic breasts have consulted us whether lifting their breasts is possible without volume reduction reduction. We explained them that lifting the nipple areola position requires some volume reduction, but we could keep it as minimal as possible. Thus we have modified the superior pedicle with verticle scar breast reduction technique due to patients’ requests and applied in 25 consecutive cases. The thickness and shape of the pedicle and the amount of resected tissue are designed to increase nipple projection and elevate its position at the same time.

## MATERIALS AND METHODS

From January 2006 to May 2012 twenty five individual patients were operated with described superior pedicle and vertical scar technique. Young women between ages 25-35 years with voluminous breasts who request mastopexy rather than reduction were selected for the surgery. Other inclusion criterion was the transposition distance of the nipple should be less than 10 cm. The medical records of the patients were documented. All patients were informed about the procedure with written and verbal information. 

Reference points such midsternal line, breast meridian, inframammary fold (IMF) were preoperatively marked on the patient in the upright position. The distance from suprasternal notch to nipple (SSN-N) was also measured. The future position of the nipple, the borders of the lateral and medial pillars was determined according to previous literature.^4^^,^^7^ Briefly, the upper border of new areola was put on the breast surface where breast meridian intersects IMF. The breast was deviated medially and laterally and pillars are marked in line with breast meridian. At this stage the drawings were differantiated slightly from classical descriptions. 

**Fig. 1:**
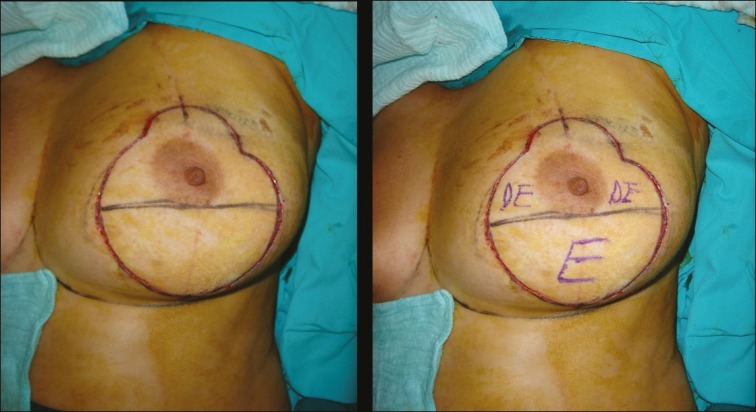
Preoperative markings. The Design of the pedicle was termed as “ Snowman Pattern”. Abbrevations: DE: Desepitelisation, E: Excision

We preferred geometrical exact shapes rather than freehand drawings. Between the medial and lateral borders of pillars an uncompleted circle was formed joining at the inferior border in rounded fashion. The distance from IMF to inferior aspect of pillar junction remained between 2-6 cm. Then areolar opening was drawn as a half circle, which circumference is equal to an areola in 42 mm diameter. Thus two intersecting circles were obtained. The pedicle to be de-epithelialized was determined by a line drawn at least 1 cm below the nipple areolar complex. The design of the pedicle and excised tissue was termed as “ Snowman Pattern” and shown in Figure 1. 

**Fig. 2:**
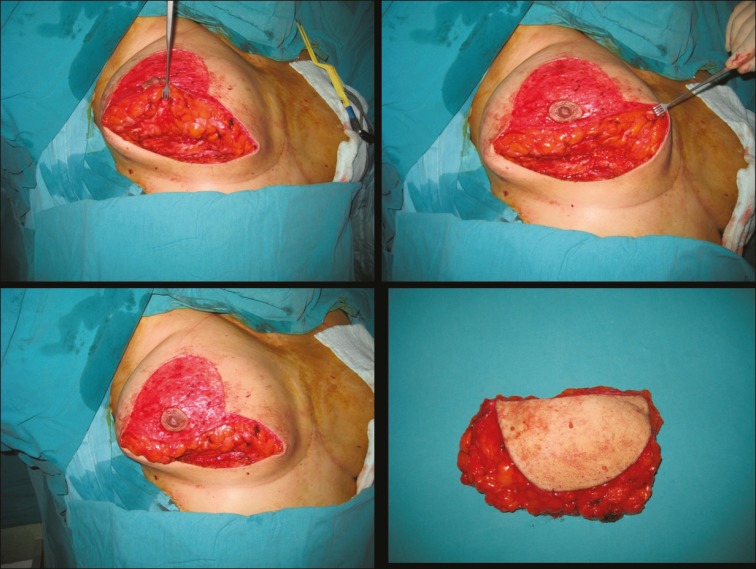
Intraoperative views of surgical procedure

All surgeries were performed under endotracheal anesthesia. No tumescent solution was administered to the breasts. At the beginning of the operation the new nipple areola complex (NAC) was outlined using a 42 mm diameter areolar marker and incised. The superior pedicle was de-epithelialized between the preoperative markings ,keeping NAC intact. The inferior border of the pedicle was incised down to the prepectoral fascia beyond the thickness of the pedicle. Lateral and medial incisons were made through the breast parenchyma in the same manner. The inferior border was elevated as a 2-3 mm thin flap down to IMF border. The inferior breast parenchyma was resected en bloc within these borders off the prepectoral fascia leaving some loose areolar tissue (Figure 2). The superior pedicle was seperated off the chest wall, creating a pouch 6-8 cm in width, till the new position of NAC, to facilitate upward movement. 

The NAC was transposed superiorly to its new location and the areolar opening was closed around it with 3/0 and 4/0 vicryl sutures. The breast thus gains better projection. After the lateral and medial pillars are approximated with 2/0 vicryl sutures, nipple projects further. The superficial layer of the vertical wound and areolar opening were closed with 4/0 absorbable sutures. The inferior part of vertical scar was gathered with “wrinkled” sutures, to prevent an inferior dog ear or extension beyond the IMF. In this technique nipple transposition more than 10 cm is avoided to prevent kinking of the pedicle and resultant vascular compromise. The full thickness superior pedicle contributes to nipple projection, although it can not be easily transposed to its new position. 

## RESULTS

**Fig. 3:**
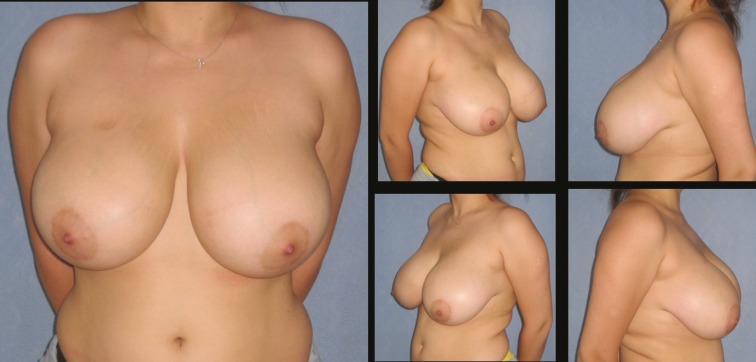
Patient-3; 25-year-old women preoperative breast views

**Fig. 4:**
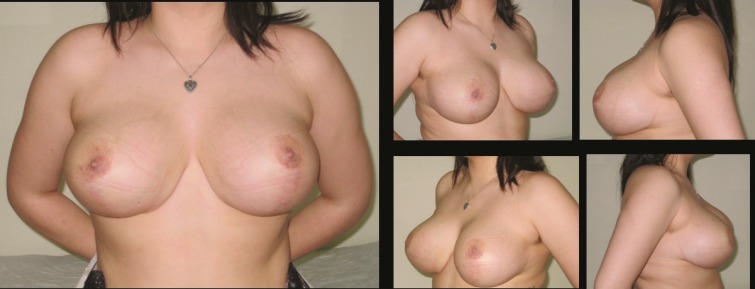
22 months postoperative result of patient-3

**Fig. 5:**
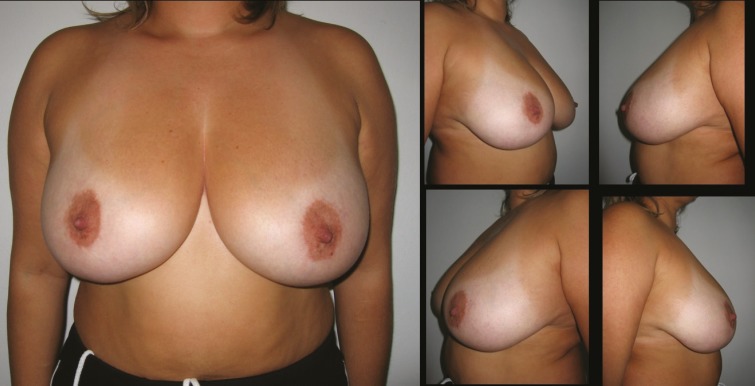
Patient-9; 35-year-old women preoperative breast views

**Fig. 6:**
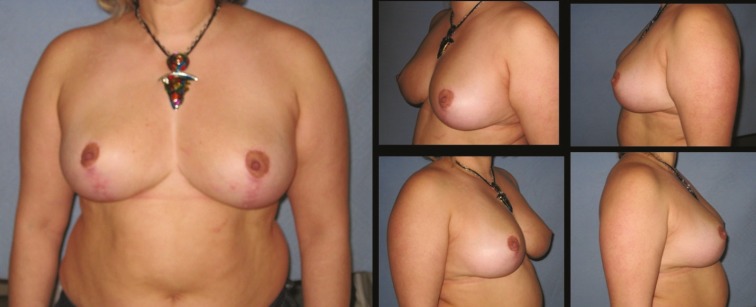
Eighteen months postoperative results of patient-9

**Fig. 7:**
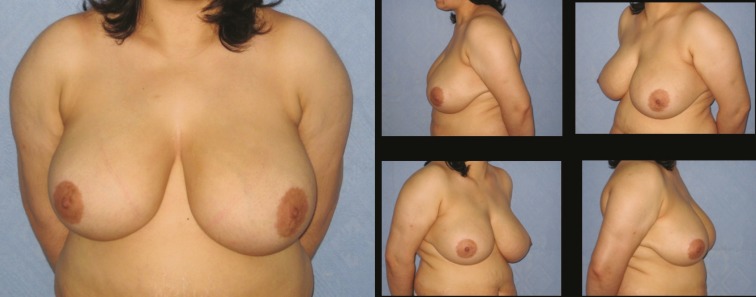
Patient-17; 35-year-old women preoperative breast views

**Fig. 8:**
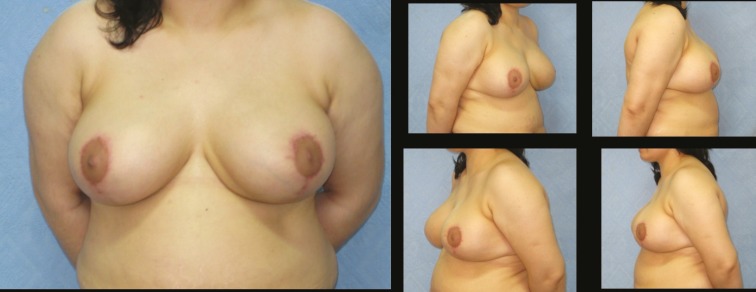
Twelve months postoperative results of patient-17

The mean patient age was 30 and body mass index (BMI) 27.8±1.07 kg/m^2^. Mean nipple transposition was 6.5 cm. Mean weight for resected tissue was 415 gr for left and 419 gr for right breast. Mean operative time was 125 minutes. Patients were followed up for 9-22 months (mean 13 months). The postoperative results of three patients are shown in figures 3-8. Demographic data is summarized in Table 1.

**Table 1:** Demographic data of the patients

**Patient No.**	**Age**	**BMI**	**Original SSN-N distance (cm) (L/R)**	**Transposed SSN-N distance (cm) (L/R)**	**Weight of resected dermoglandular tissue (g) (L/R)**
1	32	26	28/28.5	21	348/367
2	28	27.2	29/29	22	450/357
3	25	26.4	28/28	20	480/465
4	33	29	32/31	22	490/442
5	31	28.2	30/30.5	22	355/390
6	34	27.8	29.5/29.5	20	448/390
7	30	26.2	26.5/27	21	268/279
8	33	28.6	31/31	22	404/442
9	35	29.2	28/28.5	21.5	352/385
10	28	27.5	30/29	22	467/369
11	32	30.1	29/29	22	432/458
12	26	29.4	26.5/26	21	389/358
13	34	28.2	30/31	22	490/498
14	29	29.3	27/28	21	408/432
15	27	28.3	29.5/30	22	468/508
16	31	29.0	30/30	22	490/453
17	33	27.8	29.5/29.5	22	420/478
18	28	26.2	27/28	21	398/452
19	26	27.5	26/25	21	298/346
20	33	28.2	29/29.5	22	442/486
21	32	27.1	28/28	22	456/430
22	29	26.5	27/27	21	346/333
23	32	28.1	30/29.5	22	480/499
24	31	27.4	27/27	21	398/422
25	29	27	28/28	22	404/430

Suprasternal notch to nipple: SSN-N; Body mass index: BMI

**Fig. 9:**
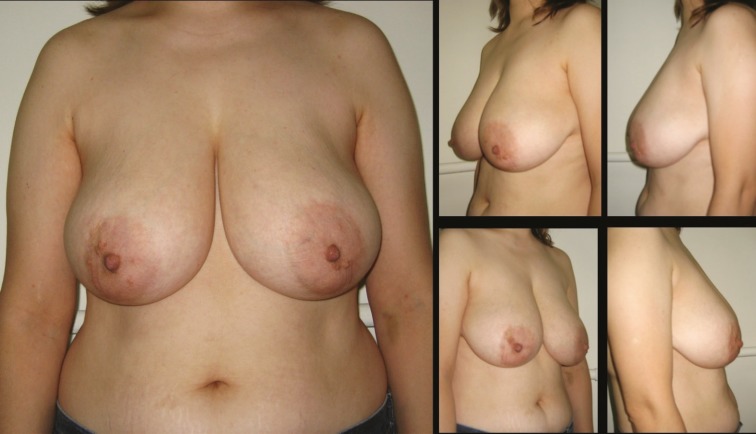
Patient 20; 33-year-old women preoperative breast views.

**Fig. 10:**
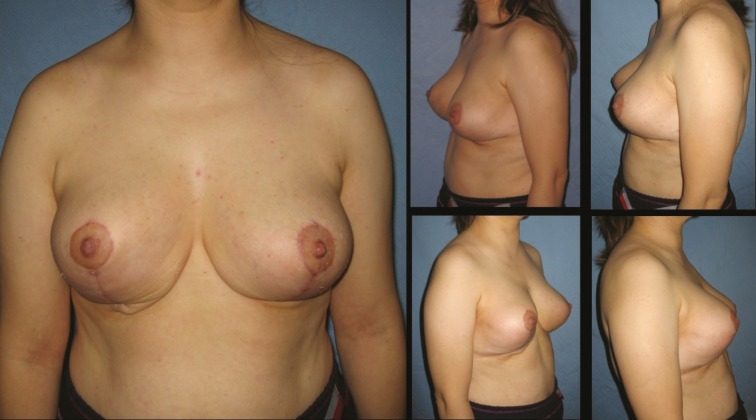
Twenty two months postoperative results of patient-20. The permenant wrinkling at the vertical scar is not resolved during the follow up period. Revison was offered to patient, she was not accepted a second intervention

No serious complications such as hematoma, infection, wound dehiscence or nipple necrosis were encountered in our patient series. This was as a result of working in a small group of patients and non-aggressive surgery. The only complication was permanent wrinkling at the the vertical scar in 5 of 25 patients probably due to vertical closure which did not resolved during the follow up period (Figures 9-10). Although revison was offered to patients, none of them accepted a second intervention. 

## DISCUSSION

Breast reduction surgery provides physical comfort and improved life quality which was diminished by Macromastia.^8^ However, most young women do not openly express such complaints and are happy with their large breasts because they feel themselves as sexually attractive. They are only dissapointed when their large breasts become “saggy” sooner. Patients may request the surgeons to surgically lift their breasts and increase projection rather than reduce them. In some patients, who are slightly overweight, less tissue resection enables more favorable results.^9^ In this situation the surgeon must focus on resecting less breast tissue than he or she usually does. Thus, we have made some modifications in the dermoglandular tissue resection pattern, pedicle design and areolar opening in superior pedicle vertical scar mammoplasty technique. 

Originally the superior pedicle is not a full thickness one like the way we elevated it for more projection. Its blood supply is superficial. Before Hall-Findlay developed her own superomedial reduction techhnique, she used superior pedicle as a full thickness pedicle similar to us. But she pointed out that, her attempts ended up with vascular compromise.^10^ However, we did not experienced any difficulty in transposing pedicle as long as we considered tissue requirements. We always performed surgery in moderate sized breasts which were required less than 10 cm transposition according to Hidalgo’s recommendation.^7^

O’Dey *et al.* stated that modifications of the superior pedicle might be considered with an SSN:N beyond 30 cm to reduce vascular complications.^11^ Whether full thickness or split thickness the superior pedicle can be used in both manner. But its blood supply, which depends mainly on perforators from higher thoracic artery and internal mammary artery is regarded as moderately reliable, when compared with other pedicles used in breast reductions.^12^ Some modifications in superior pedicle vertical scar mammoplasty have been proposed to increase pedicle length while preserving vascularity.^13^

However, using this technique in large breasts was not widely accepted. Due to unfavorable results most surgeons do not perform this method in major reductions.^9^ If the superior pedicle is attempted to transpose to higher levels in large breasts, the pedicle must be thinned up to 1-1.5 cm for easy insertion into areolar opening at the new nipple position.^14^^,^^15^ Although, thinning allows easy folding and insertion, aggressive resection of the deep portion of the superior pedicle can produce a poor projection in newly forming breast.^7^

In this circumstance the surgeon has to favor other pedicles independent of the type of resulting scar. Bivectorial superior pedicle is a suitable option even for long pedicle, while it creates a natural breast profile and projection, additionally decreasing tension on NAC.^5^ But dissection should be carried out carefully with regard to vascular anatomy. Superior pedicle can be replaced by superomedial pedicle, if the areola is not transected by a line between the endpoint of areolar opening.^16^ In heavy hypertrophy with lower nipple levels, inferior pyramidal pedicle enables easier transposition and more tissue resection.^17^ Pedicle decision making for breast reduction is out of the scope of this article. We focus only on using superior pedicle in small reductions with short scars, due to patients’ requests. A novel technical modification for superior pedicle reduction is described based on mastopexy limited inferior pole resection in slightly ptotic large breasts. 

In the present article instead of the classical dome pattern “snowman’’ drawings are described. This modification facilitates surgical planning and learning due to its geometrical design. It contains two circles with different diameters transecting each other. The smaller circle is employed as an areolar opening whereas the bigger includes dermoglandular tissue to be reduced. More experienced surgeons prefer artistic manipulations rather than measurements.^18^ But for less experienced surgeons accurate technical instructions are more valuable. Snowman design which sometimes was tried to be standardised with templates is more exact than free hand drawings.^15^^,^^19^

It enables to increase projection without relocating nipple to a higher position. Because pedicle transposition movement is more controlled, it allows horizontal tissue resection thereby minimising tissue resection. As the areolar opening was closed, a more pojectile breast with upper pole fullness was obtained. As noted previously by Hidalgo, if smallest possible circumference is chosen as an open design, greater the flexibility in determining final nipple position. This also reduces the risk of hypertrophic circumareolar scar.^7^

The vertical scar mammoplasty promises the surgeons and patients a better shape and projection, shorter scars and long-lasting results.^20^ However, complication rates varied between the studies.^21^^-^^23^ Complications such as hematoma, seroma formation, scarring, wound dehiscence can be minimized with limited amount of undermining and liposuction.^23^ We have not experienced any complications mentioned above except wrinkling at the the vertical closure line. With appropriate indications vertical scar breast reduction is a good choice with a higher patient satisfaction.^24^^,^^25^ Technical refinements and simplification of the procedure would decrease complications and make this technique more surgeon friendly. 

Usually, the vertical scar is closed by subcuticular wrinkled sutures which resolve over time.^26^^,^^27^ This type of closure helps to decrease the height of vertical scar. However, the only unfavorable result seen in our patients is the permanent wrinkling caused by purse-string closure especially vertical scar, which was not resolved in five cases during the follow-up period. This situation can be attributed to our more rounded skin excision design, which loads more tension onto the skin than conventional vertical scar breast reduction methods. 

The wrinklings at the IMF have not been felt as a serious problem by our patients. Even we have recommended them surgical revision by converting vertical scar to a short inverted T scar or liposuction near IMF,^3^^,^^28^ none of them has accepted revision . Refusal of revisions of our patients are correlated with the previous literature, which showed that short scar options would be preferred by women considering breast reduction surgery.^24^^,^^29^ It is demonstrated that in comparative studies vertical scar versus inverted T, the complication rates were found similar.^24^^,^^25^ Mostly women are apprehensive about long unsightly scars, if correction of ptosis is their primary concern. Thus the importance of developing and perfecting techniques that minimize scars in breast reduction is experienced again in our method. 

Independent management of skin and gland is helpful in decision making of the right technique for each individual patient based on geometrical concepts.^30^ Provided that the described technique is regarded as a mastopexy and projection enhancement procedure, it can not be adapted for atrophied and severely sagging breasts. The special indication for this technique is young women who have voluminous but ptotic breasts and request lifting rather than reduction. 

Many options are recently available in the era of modern breast reduction surgery. We recommend that our “snowman design” is a useful tool for superior pedicle breast reduction techniques providing a good projection and short scar, in selective patients. 

## CONFLICT OF INTEREST

The authors declare no conflict of interest.

## Note

Please cite this paper as:

Cil Y, Kocman AE. A New Reduction Mastopexy Design for Young Women: Snowman Pattern. World J Plast Surg 2015;4(2):110-119.
